# Implementation of a deep learning model for automated classification of *Aedes aegypti* (Linnaeus) and *Aedes albopictus* (Skuse) in real time

**DOI:** 10.1038/s41598-021-89365-3

**Published:** 2021-05-10

**Authors:** Song-Quan Ong, Hamdan Ahmad, Gomesh Nair, Pradeep Isawasan, Abdul Hafiz Ab Majid

**Affiliations:** 1 UOW Malaysia KDU Penang University College, 32, Jalan Anson, 10400 George Town, Pulau Pinang Malaysia; 2 School of Computer Sciences, Universiti Sains Malaysia, Penang, Malaysia; 3 Vector Control Research Unit, School of Biological Sciences, Universiti Sains Malaysia, 11800 Penang, Malaysia; 4 Faculty of Computer and Mathematical Sciences, Universiti Teknologi MARA, Perak Branch, Tapah Campus, 35400 Tapah, Malaysia

**Keywords:** Computational biology and bioinformatics, Zoology

## Abstract

Classification of *Aedes aegypti* (Linnaeus) and *Aedes albopictus* (Skuse) by humans remains challenging. We proposed a highly accessible method to develop a deep learning (DL) model and implement the model for mosquito image classification by using hardware that could regulate the development process. In particular, we constructed a dataset with 4120 images of *Aedes* mosquitoes that were older than 12 days old and had common morphological features that disappeared, and we illustrated how to set up supervised deep convolutional neural networks (DCNNs) with hyperparameter adjustment. The model application was first conducted by deploying the model externally in real time on three different generations of mosquitoes, and the accuracy was compared with human expert performance. Our results showed that both the learning rate and epochs significantly affected the accuracy, and the best-performing hyperparameters achieved an accuracy of more than 98% at classifying mosquitoes, which showed no significant difference from human-level performance. We demonstrated the feasibility of the method to construct a model with the DCNN when deployed externally on mosquitoes in real time.

## Introduction

Dengue fever threatens over one-third of the world’s population and causes 100 million dengue infections worldwide every year^1^. With no licensed vaccines or specific treatment, the control of the disease depends greatly on the control of the vectors: *Aedes aegypti* (Linnaeus) and *Aedes albopictus* (Skuse). Previous studies^2^ showed that the insecticide susceptibility patterns of *Ae. aegypti* and *Ae. albopictus* were not homogeneous across geographic regions, and the control strategy, such as the selection of an insecticide’s active ingredient, depends on the result of mosquito monitoring since *Ae. aegypti* and *Ae. albopictus* have different distributions, breeding habitats, etc. To monitor mosquitoes, field sampling of larvae or adult mosquitoes is commonly conducted, and later, the species are classified in a laboratory^3^. However, sorting mosquitoes relies mainly on the manual examination of the morphological keys and taxonomic characteristics of mosquitoes, which is challenging, especially for *Ae. aegypti* and *Ae albopictus,* which share many similarities. Human expert recognition usually focuses on conspicuous patterns on the dorsal thorax formed by white scales. *Ae. aegypti* typically has white, lyre-shaped markings, whereas *Ae. albopictus* has a median-longitudinal white stripe^4^, but the task becomes more demanding when the features disappear, especially on mosquitoes collected from the field.

The development of a computational model using artificial intelligence (A.I.), especially machine learning (ML) algorithms, provide an excellent alternative for mosquito recognition and classification. Numerous studies have used different properties of mosquitoes as labeled data to train and construct ML models. For example, Silva et al.^5^ applied audio analysis techniques and different ML algorithms to recognize *Ae. aegypti*, *Anopheles gambiae* Say, *Culex quinquefasciatus* Say and *Culex tarsalis* Coquillett and obtained an accuracy of 50.91% to 87.33%. De Los Reyes^6^ proposed the recognition of *Aedes* mosquitoes by using digital image processing techniques with a handcrafted feature-based method and achieved a maximum accuracy of 87.41%. Mulchandani et al.^7^ used wingbeats as the spectrogram input and compared different pretrained computational models for the identification of *Ae. aegypti*, *Ae. albopictus*, *An. arabiensis*, *An. gambiae*, *Cu. pipiens* L. and *Cu. Quinquefasciatus*. They reached a maximum accuracy of 86%. However, the data acquisition steps of these previous works involved considerable manual feature extraction and large quantities of data/images, making the approaches laborious and time consuming and unable to be performed in real time. Deep convolutional neural networks (DCNNs) of deep learning (DL) are state-of-the-art methods for object recognition and classification, including for agricultural pests and mosquito larva^8,9^. With feature extraction in the neural network layers, DL has a high potential to make the development of a model easier and more accurate^10^.

Another major bottleneck in the classification of mosquitoes is the condition of the samples. In reality, especially for mosquitoes collected from the field, the markings on the dorsal thorax commonly have some level of disappearance^11^, which increases the difficulties of recognizing and distinguishing closely related interspecies, such as *Ae. aegypti* and *Ae. albopictus,* that share significant morphological similarities. The issue was not focused on by the previous studies that came from the perspective of computer science, in which a dataset was mostly constructed by taking data from the internet (via data mining)^6,12^ or images of the mosquito species that were in good condition^10,13,14^, which may make the model impractical when deployed on actual samples. Motta et al*.*^15^ approached this issue by constructing a dataset with field-collected *Ae. aegypti*, *Ae. albopictus* and *Cu. quinquefasciatus* mosquitoes but only achieved an accuracy of 83.9% for *Ae. aegypti* and *Ae. albopictus*. Park et al*.*^16^ applied a more systematic approach by training the model with mosquitoes with different deformations and managed to obtain a higher accuracy of 97% in the classification of three genera of mosquitoes.

In this study, we built a piece of hardware—the Aedes Detector—that can regulate the process of image acquisition, training, validation and testing for a DL model. Our proposed model utilized a web-based tool that uses transfer learning with a DCNN, which allows the model to be externally executed in real time, and the web tool and transfer learning make it possible, to work in real time. The objectives of this study are as follows.

### Convenient approach to developing a model

Image-based classification usually requires considerable domain expertise to design the feature extractor for the images, and common learning algorithms require large datasets and excessive amounts of central processing unit (CPU) power^17–19^. Therefore, we demonstrated the capability of a web-based tool from Google Creative Lab—Teachable Machine 2.0—that conducts image acquisition and trains a computational model with no coding required.

### Datasets of *Ae. aegypti* and *Ae. Albopictus*

Although the issue of imperfect mosquito samples was addressed by some previous works^10,13,15^, the datasets consisted of relatively fewer images of *Ae. aegypti* and *Ae. albopictus* that were older than 12 days, especially the white scales on the thorax that were disappeared. Therefore, one of our goals for this study was to build a dataset with a total of 4,120 images that consisted of mosquitoes of older age and different levels of head and thorax scale disappearance.

### Transfer learning and hyperparameter analysis

Training a state-of-the-art DCNN such as MobileNet and AlexNet requires a large quantity of data^19^, and 4,120 images from our experiment were not enough to build the DCNN. We applied the transfer learning technique in which the weights of the layers from the DCNN were transferred to recognize the mosquitoes. Teachable Machine 2.0 allows the pretrained DCNN models to be optimized with two major hyperparameters—the number of epochs and the learning rate (LR).

### Hardware implementation of the model and human-level performance

Despite numerous studies demonstrating the ability of a DCNN to provide 80 to 97% accuracy to classify medically important mosquitoes^7–11,15,16^, these results that use a large pretrained model might not be practical when deployed in hardware/devices. We deployed the proposed model in a platform called p5.js visual coding tool written in JavaScript by using the microcomputer of the Aedes Detector to verify the proficiency of the model at classifying a mixture of three different generations of mosquitoes in real time. By using the same dataset, the accuracy of deployment ability was compared with the human experts’ performance.

## Methods

### Acquisition and splitting of data

**Figure 1:**
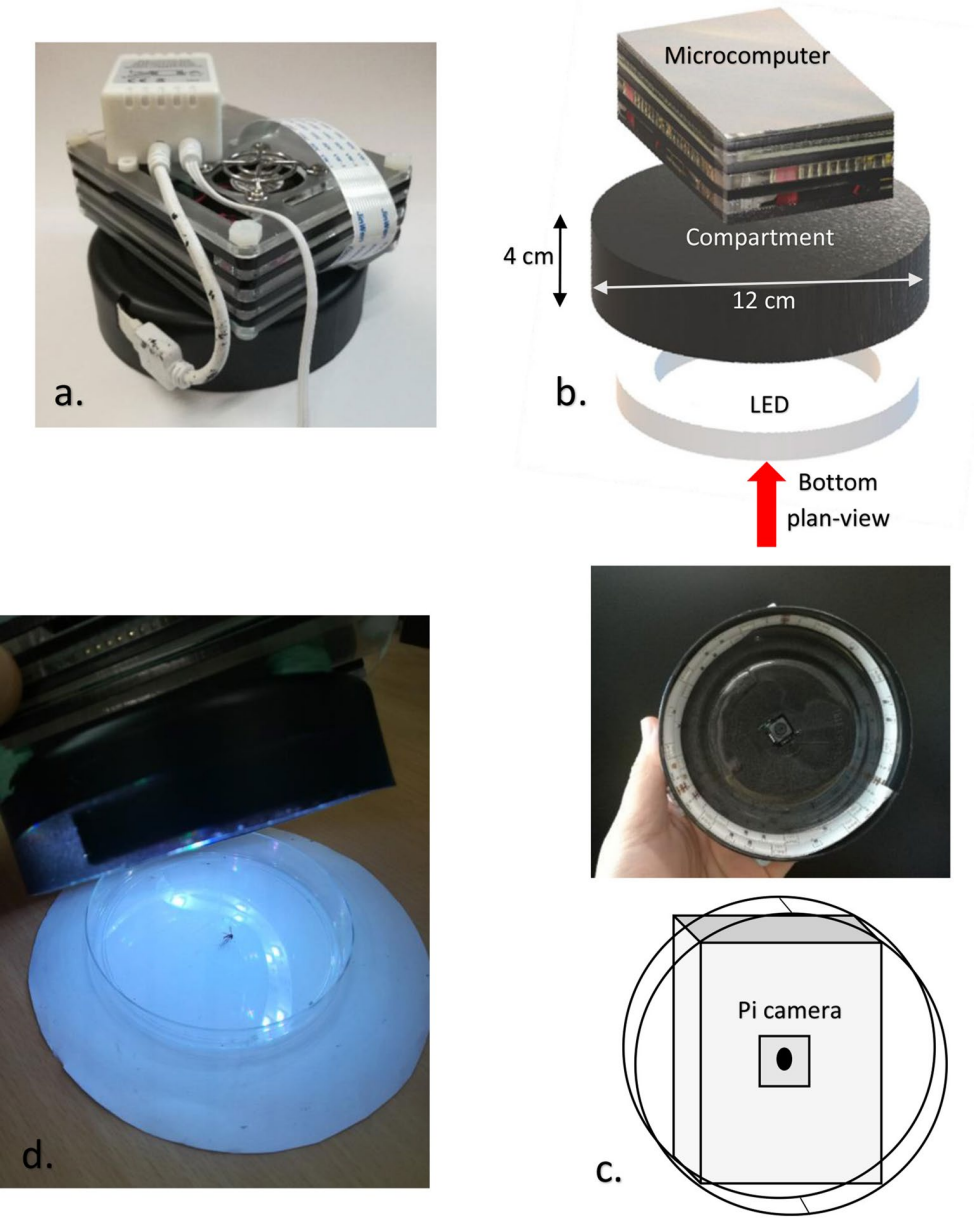
Aedes Detector: (**a**) actual device demo; (**b**) 3-dimensional isometric projection of the device with the components and dimensions; (**c**) bottom plan-view and the position of the Pi camera; (**d**) operation of the device with a sample at the center of the Petri dish.

**Figure 2:**
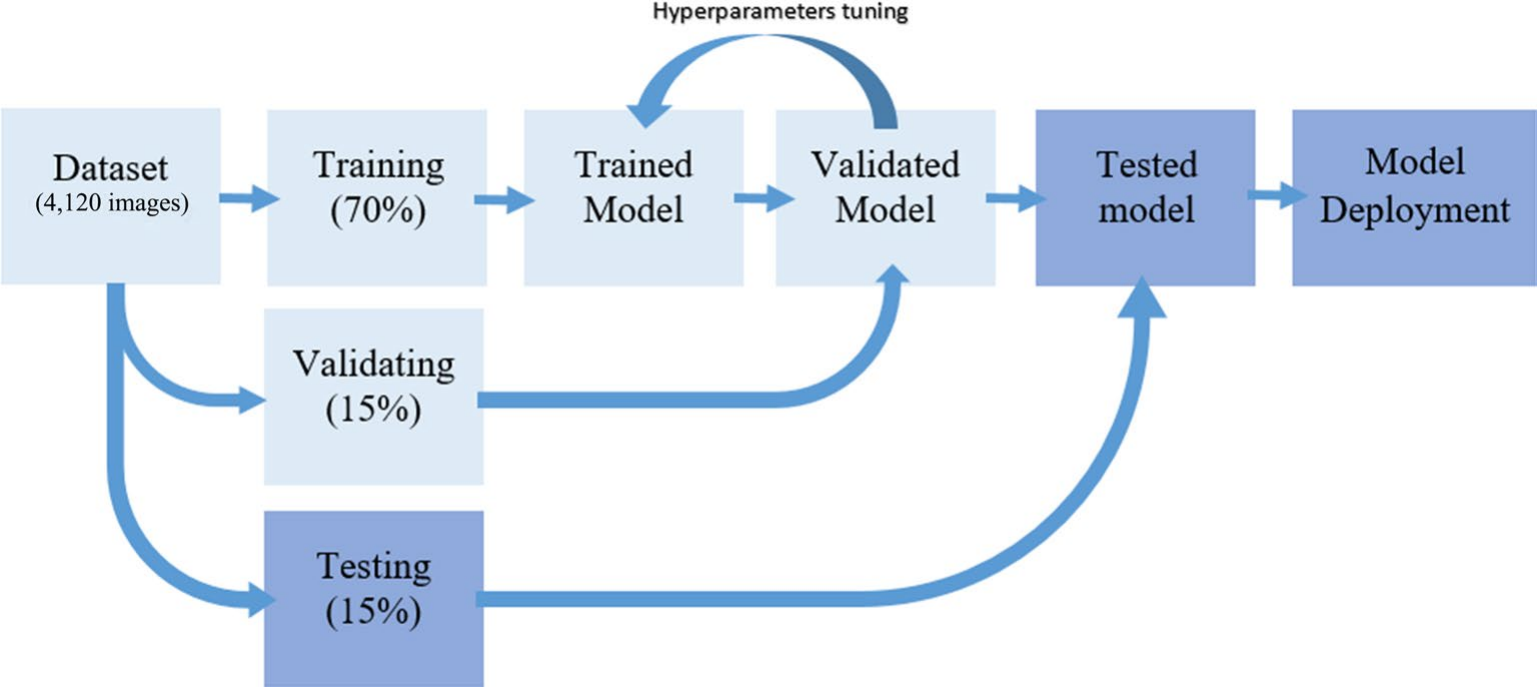
Data splitting and process to be used for training, validating and testing the model. The dataset of 4,120images is split into 70% training (2,884 images), 15% validation (618 images) and 15% testing 618 images).

Previous works^5–11^ reported limited information, such as the focal length, background, and illumination (wavelength and intensity), when acquiring data. To address the problem, we built a piece of hardware called the Aedes Detector to improve the image acquisition, training and testing processes of the model. The construction of the Aedes Detector is detailed in Fig. 1. The device was equipped with a microcomputer (Raspberry Pi 4 Model B, Quad-core Cortex-A72, and 2 GB LPDDR4-3200 SDRAM) with a black cylindrical compartment (diameter of 12 cm and height of 4 cm), which provided a short focal length (2.5 cm), illumination (15 white-colored LEDs—RGB visible light) and background (white color). The approach we used in this study aimed to serve as a framework that can be conveniently applied by other experts, especially entomologists. To investigate and be able to fine-tune the computational model for insects, we constructed the proposed model by using Teachable Machine 2.0 (Google Creative Lab) that worked on the MobileNet DCNN^20^, which consisted of a feature extractor for the mosquito images from the ImageNet dataset. The mosquito images were acquired by a camera module (Pi NoIR v2, 8 megapixels, Sony IMX219 image sensor) on the Aedes Detector. To avoid overfitting this model, data augmentation was performed on the training dataset. All images of the dataset (training, validation and testing) were rotated by 0, 90, 180, and 270 degrees; thus, eventually, the number of samples was increased by four times. The data splitting/partitioning used for training, validating and testing the model is illustrated in Fig. 2, which summarizes how the data partitioning was carried out by leveraging the platform of Teachable Machine 2.0—Training (phase 1—70%), validating (phase 2—15%)^21^, and evaluating the testing dataset (phase 3—15%). The base images (0 degree, without rotation) and all the rotated images (90, 180, and 270 degrees) used for training were not used for validation and testing set. The model was developed to classify the mosquitoes into three different classes according to *Ae. aegypti*, *Ae. albopictus* and background (with no sample), and a total of 4,120 images (2,040 per species and 40 for background) were used in this study. Synthetic minority oversampling technique (SMOTE) was used to tackle the imbalance of the class of background, in which random oversampling was conducted to duplicate the images of background in training set.

### Deep convolutional neural network (DCNN)

#### DCNN architecture

**Figure 3:**
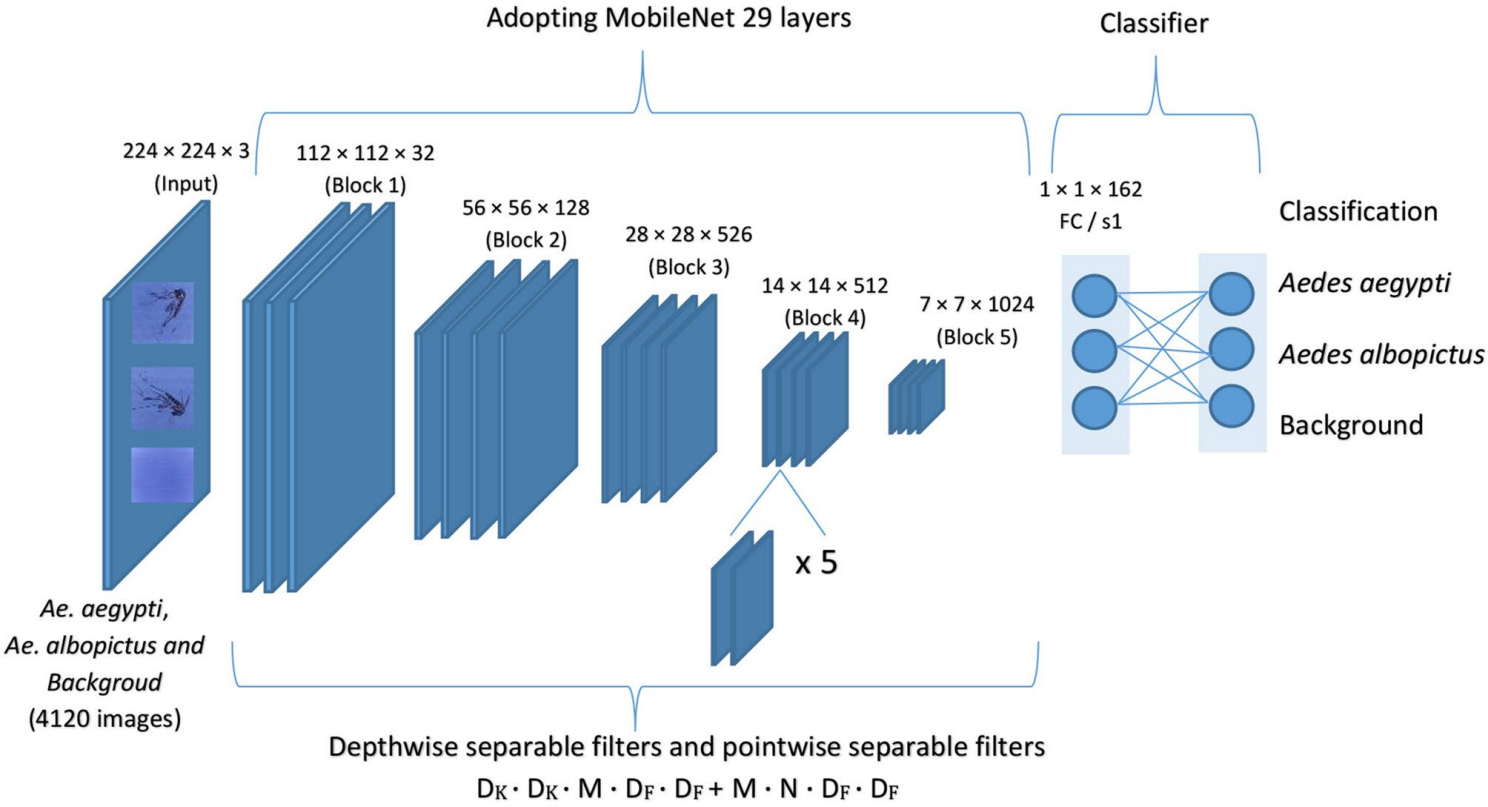
The workflow of MobileNet DCNN architectures and transfer learning model of the present study.

The proposed model was based on the Teachable Machine 2.0—DCNN architecture of MobileNet that transfers the learning weights to reduce the training time, mathematical calculations and the consumption of the available hardware resources^22^. The workflow of the classification of Teachable Machine 2.0 is shown in Fig. 3. The first 29 convolutional layers of MobileNet were adopted^23^, which were used to extract the features. More convolutional layers can reduce the resolution of the feature map and extract more abstract high-level features^24^. The softmax layer of MobileNet was truncated, and the output of the model was set as the last tensor representation of the image. Therefore, the web-based platform allowed us to create two layers—the first dense layer and the final softmax layer—with three classes (images of *Ae. aegypti*, *Ae. albopictus* and background). The first dense layer must take the same input as the output of MobileNet, and the transformation of the data to their tensor was performed by MobileNet.

#### Training DCNN with hyperparameter analysis

In earlier studies, most of the attempts involving the classification of *Ae. aegypti* and *Ae. albopictus* using dynamic hyperparameters demonstrated a maximum accuracy of 84–87%^10,13,15^. Since deep learning neural networks are trained using the stochastic gradient descent algorithm, hyperparameters such as the learning rate (small positive value ranging from 0.0 to 1.0) that controls the rate of the change to the model during each step of the optimization process have to be determined based on the particular dataset^25^. Instead of training the model from scratch, and since the number of epochs and learning rate are available in the platform—Teachable Machine 2.0—we aimed to obtain a best-performing model by analyzing the effects of the number of epochs and the learning rate (LR) and their interactions. We standardized the batch size to 16, and two-level factorial analysis (two-way ANOVA) was performed by using SPSS 17.0. The LR was started from 0.001 on a logarithmic scale. The independent factors were set, namely, seven different numbers of epochs (20, 30, 40, 50, 60, 70, and 80) and four different LRs (0.01, 0.001, 0.0001, and 0.00001), in response to the test accuracy and loss. A post hoc test was carried out on the dependent factors using Tukey’s test at p < 0.05 in SPSS 17.0, where there were significant differences in those factors.

### Hardware implementation and human-level performance

The proposed model was exported as JavaScript, and the model was uploaded through a link and deployed on the p5.js platform. The p5.js was configured to provide the prediction on a canvas (600 × 600). To infer the performance of the deployed model in real time, although Raspberry Pi camera module was reportedly to obtain 90 fps for video recording, but to prevent screen tearing, we set the frame rate as 60 fps by calling framerate (60) syntax in p5.js. To avoid the bias of overlapping data, the model deployment ability was conducted on three different batches of mosquitoes (n = 3, each batch consisted of 100 mosquitoes) that were independent of the images of the dataset used in the process of model development. The sample (randomly on a mixture of *Ae. aegypti* and *Ae. albopitus* with the number of samples = 50:50) was placed in a Petri dish in the Aedes Detector (Fig. 1), and the outputs from the p5.js canvas were recorded as the result of inference on a computer monitor in real time. The experiment was repeated by an evaluation of human-level performance, in which we invited 30 senior entomologists (12 females and 18 males, age 32–60, mean age of 46 years, consisting of more than three years of experience with insect taxonomy) to identify the images of *Ae. aegypti* and *Ae. albopictus* from the testing subset, in which the images were randomly mixed and selected from the pool of the testing subset. The images were presented to the senior entomologist via online quiz (https://forms.gle/s28ckNBtfkjoanFBA), and the accuracy was based on the percentage of the answer that correctly identified the mosquitoes. Independent-sample t-tests were performed to evaluate the accuracy between DL model deployment and human performance at p < 0.05 in SPSS 17.0. Senior entomologists voluntarily participated in the experiments, and informed consent was obtained prior to participation. All experimental methodologies were approved by the ethical committee of UOW Malaysia KDU Penang University College. All experiments were carried out in accordance with the guidelines of the ethical committee of UOW Malaysia KDU Penang University College.

## Results

### Classification performance of the DCNN

**Figure 4:**
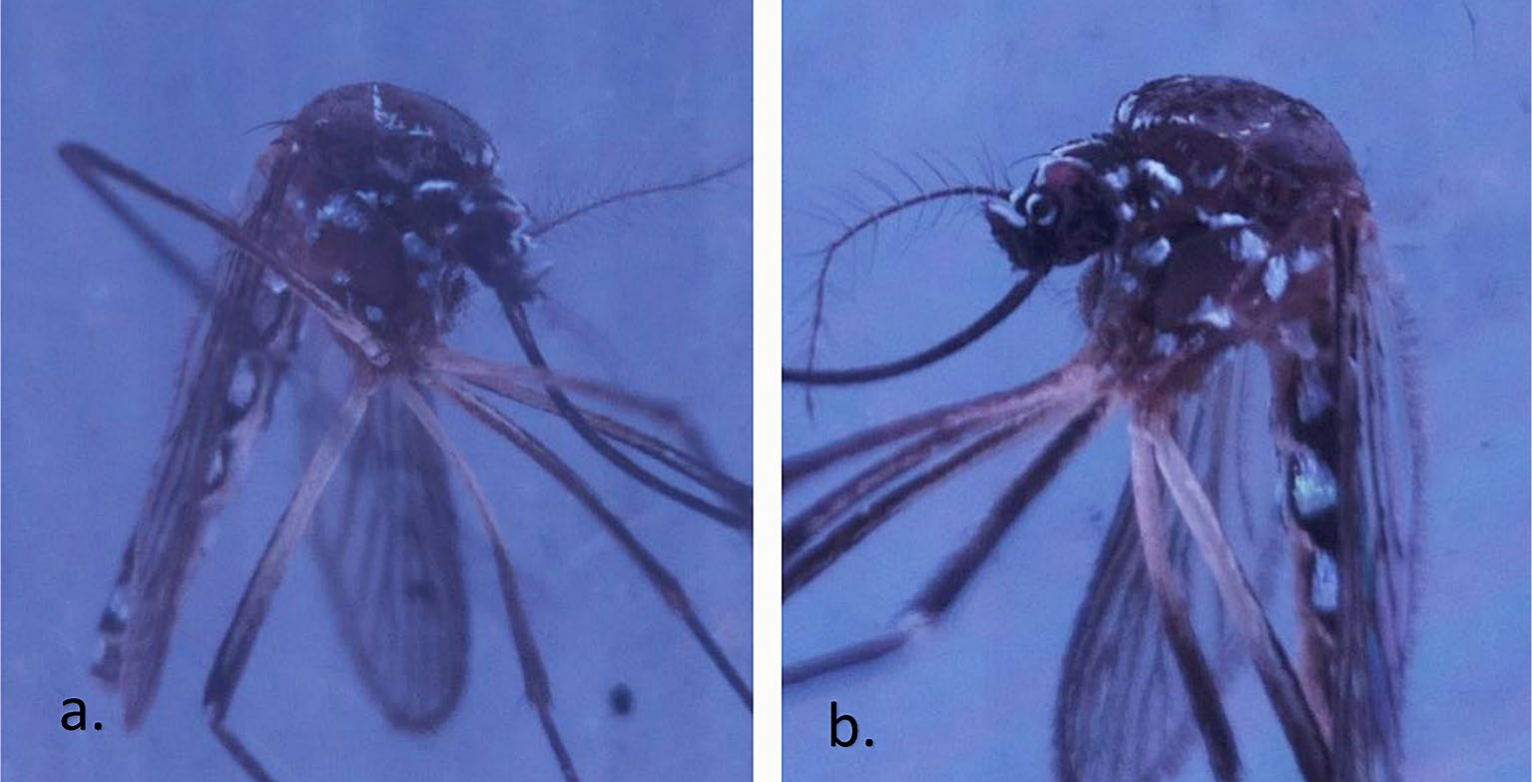
Sample of 10-day-old (**a**) and 16-day-old (**b**) *Aedes aegypti*, respectively.

**Figure 5:**
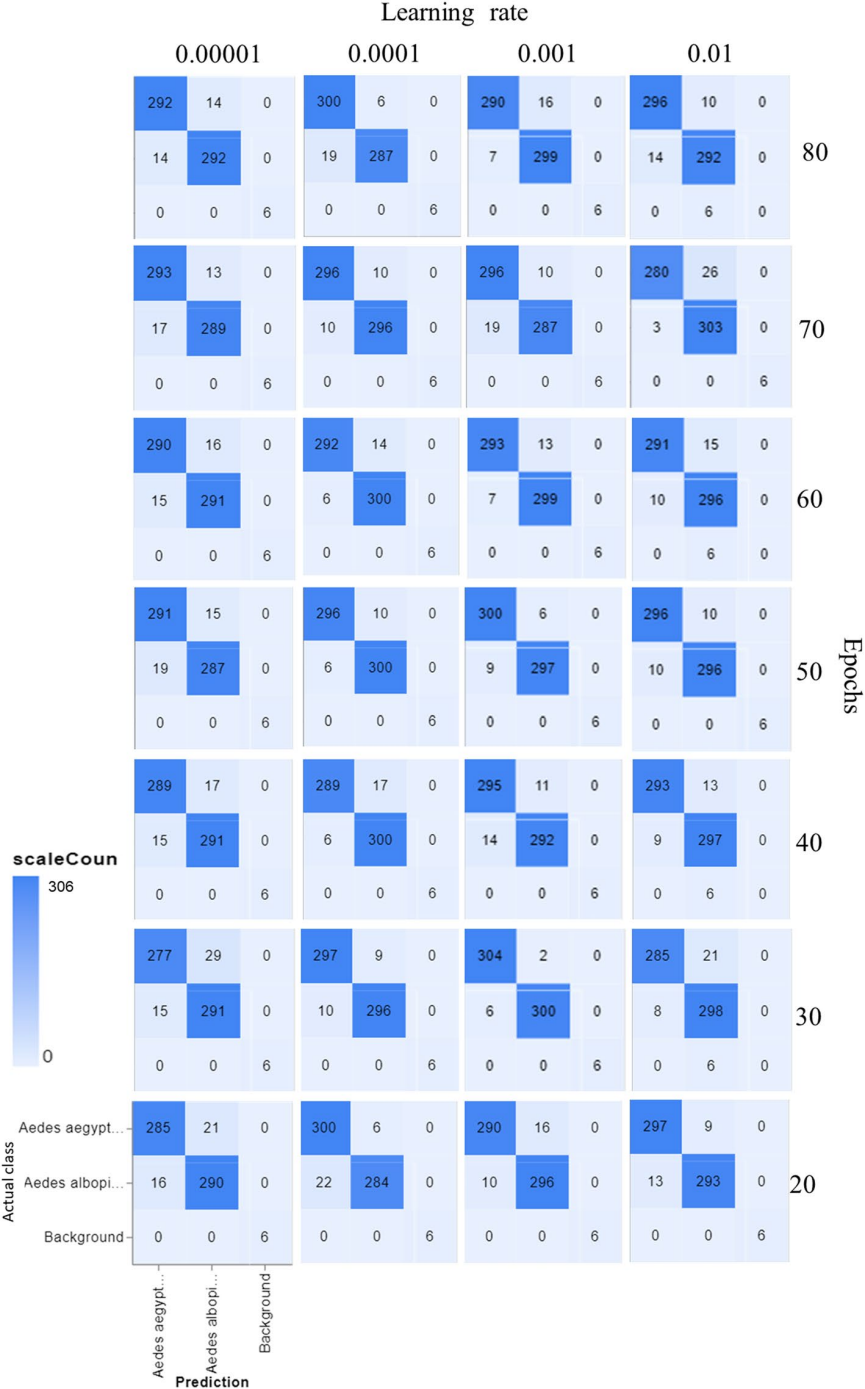
Confusion matrix of the proposed model with different epochs (vertical) and learning rate (horizontal): For the individual confusion matrix, the y-axis "actual class" refers to the ground-truth labels in the dataset, and the x-axis "prediction" refers to the model's predictions.

**Figure 6:**
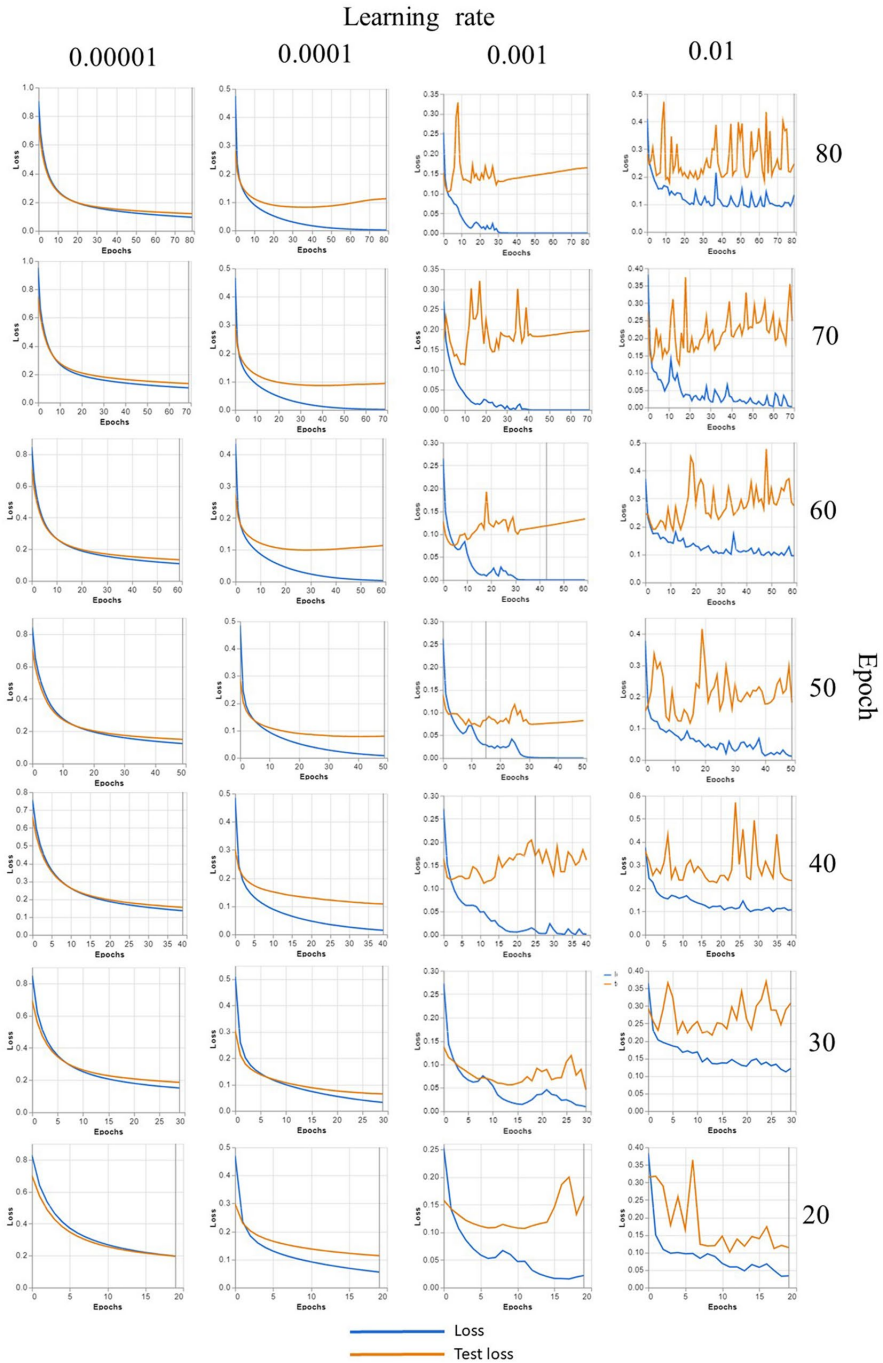
Accuracy and loss curve for epoch 30 that provides the highest accuracy. As seen in the figure, the learning curve experienced greater fluctuation/noise when we used a larger learning rate (LR).

Figure 4 shows examples of 10- and 16-day-old *Ae aegypti*, respectively, in which the lyre shapes of the white scales on the thorax were barely observed; therefore, to allow the dataset to practically reflect samples of various ages, we constructed the dataset by using female mosquitoes that were older than 12 days old. The overall test accuracy was reported in Table 1. Our results showed that both the number of epochs and the learning rate (LR) exhibited significant effects on the testing accuracy (p < 0.05), and when the number of epochs was 30 and the LR was 0.001, the model achieved an accuracy of 98.06 ± 1.02%. The number of epochs was the number of times that the entire training dataset was used for the algorithm learning process, and each epoch consisted of one or more batches that were used to tune the internal model parameter^26^. Theoretically, more epochs should result in higher accuracy^27^, although a longer runtime was required. However, from our results, more epochs did not necessarily guarantee better accuracy. This was demonstrated at an LR of 0.001, and there was a significantly lower testing accuracy from 50 to 80 epochs (Table 1). Figure 5 shows the confusion matrix for the precision and recall of the proposed DL model with different hyperparameters fine-tuned. Loss is a number representing how unfit a model's prediction is on a single example^28^, and the impacts of the number of epochs and the LR on the loss are shown in Fig. 6. As shown in Fig. 6, the learning curve experienced greater fluctuation/noise when we used a larger learning rate (LR), and having a similar trend as the testing accuracy, the learning rate (LR) significantly impacted the loss (p < 0.05), but there was no significant difference as the number of epochs changed.

**Table 1:** Hyperparameter analysis of testing accuracy (%, means ± S.E.) of the proposed model.

Epoch (F = 7.049, df = 3,6)	Learning rate (F = 16.716, df = 3,6)			
	0.00001	0.0001	0.001	0.01
20	93.30 ± 0.59a	92.68 ± 1.64a	92.73 ± 1.41a	93.75 ± 1.36a
30	92.61 ± 1.64a	96.17 ± 0.47b	98.06 ± 0.32c	93.78 ± 0.40a
40	94.15 ± 0.48a	95.04 ± 0.62a	97.14 ± 0.70b	95.14 ± 0.22a
50	93.92 ± 0.29a	96.17 ± 0.66b	96.93 ± 0.37b	95.85 ± 0.46c
60	94.26 ± 0.48a	96.55 ± 0.30b	96.39 ± 0.19b	94.22 ± 0.47a
70	94.66 ± 0.59a	95.75 ± 0.54a	96.28 ± 0.49a	95.17 ± 0.38a
80	94.64 ± 0.42a	95.27 ± 0.57a	95.80 ± 0.36a	94.91 ± 0.29a

Means ± S.E followed by same letter indicates not significantly different at P = 0.05 within row, post hoc test, Turkey. Epochs*Learning rate (F = 2.525, df = 18).

*SE* Standard Error.

### Deployment model and human-level performance

**Figure 7:**
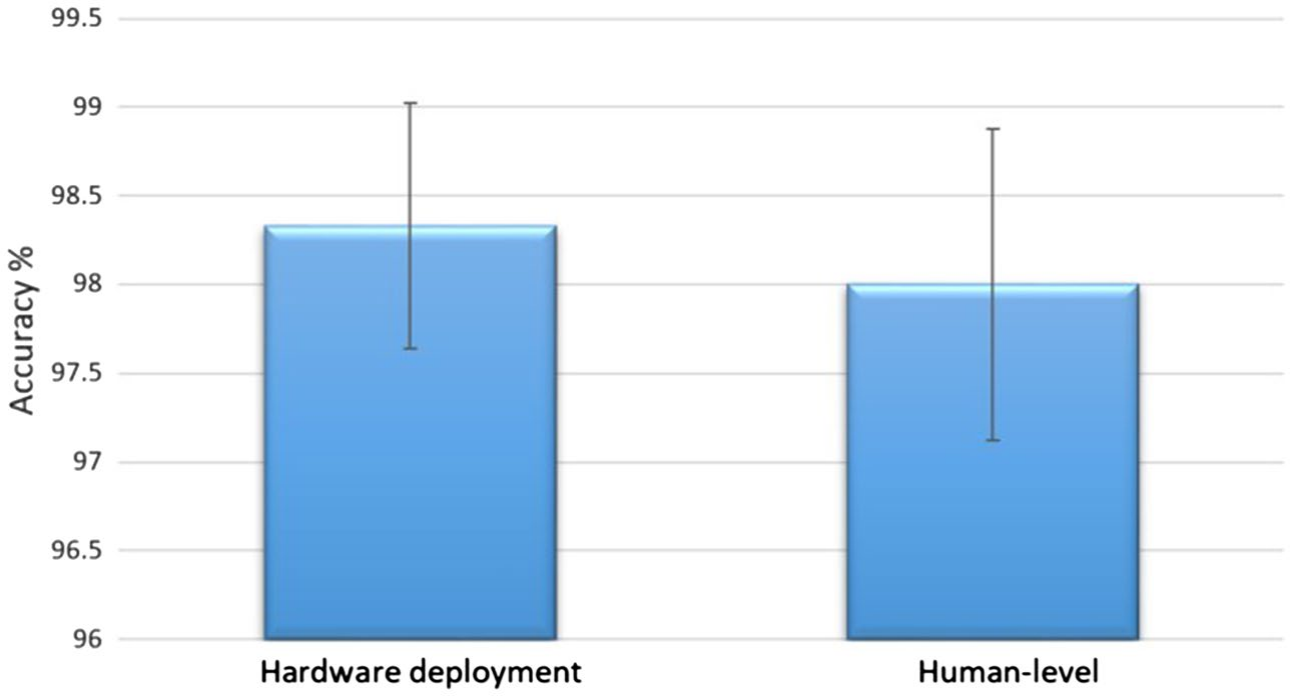
Model deployment and human-level performance accuracy as a percentage (%) of total mosquitoes used for prediction; there was no significant difference between the accuracy of hardware and human-level performance t (58) = 0.297, p = 0.768, by the independent-samples t-tests in SPSS 17.0.

For the proposed DCNN model that was implemented in a hardware tool and tested externally on different generations of mosquitoes, other factors, such as the compatibility of the model with the hardware and the portability of the model, affected the performance. Compared with previous studies^10,16,29^ that used transfer learning from other DCNNs such as VGGNet and AlexNet, MobileNet is a pretrained model with a smaller size^22,23^ that allowed our proposed model to be deployed directly in the p5.js platform using JavaScript, and the inferences were obtained in real time—60 fps (by calling the function frameRate (60) in p5.js). To the best of our knowledge, this was the first time that a DCNN model was implemented in an application platform on a hardware tool and tested externally on different generations of mosquitoes. The results of the model’s deployment and human-level performance are presented in Fig. 7. When the DL model was deployed in p5.js on the Aedes Detector for the randomized mixture of *Ae. aegypti* and *Ae. albopictus*, the DL model obtained an accuracy of 98.33 ± 0.69%. When the experiment was repeated with human performance, the senior entomologists achieved an accuracy of 98.00 ± 0.88%. Although the performance of the DL model slightly surpassed the human performance, the independent-samples t-tests showed that there was no significant difference between the accuracy of hardware and human-level performance t (df = 58) = 0.297, p = 0.768*,* indicating that the DL model performance resembled that of human experts.

## Discussion

The feature presentation of the images is crucial since MobileNet uses the k-nearest neighbor (kNN) algorithm that uses the semantic information represented in the logits to compare the images in the dataset and unknown samples as the classifier^23^. To emphasize the morphology of mosquito’s features on the mosquito images, we standardized the environment for model development (training, validating and testing) to minimize errors such as uneven and inconsistent light distributions in the sample and focal distances that make the sizes of the samples in the images different. To prevent possible overfitting of the model due to the standardized images/samples throughout the development process, the mosquito images in the dataset were manually checked to confirm no overlap among the subsets.

**Figure 8:**
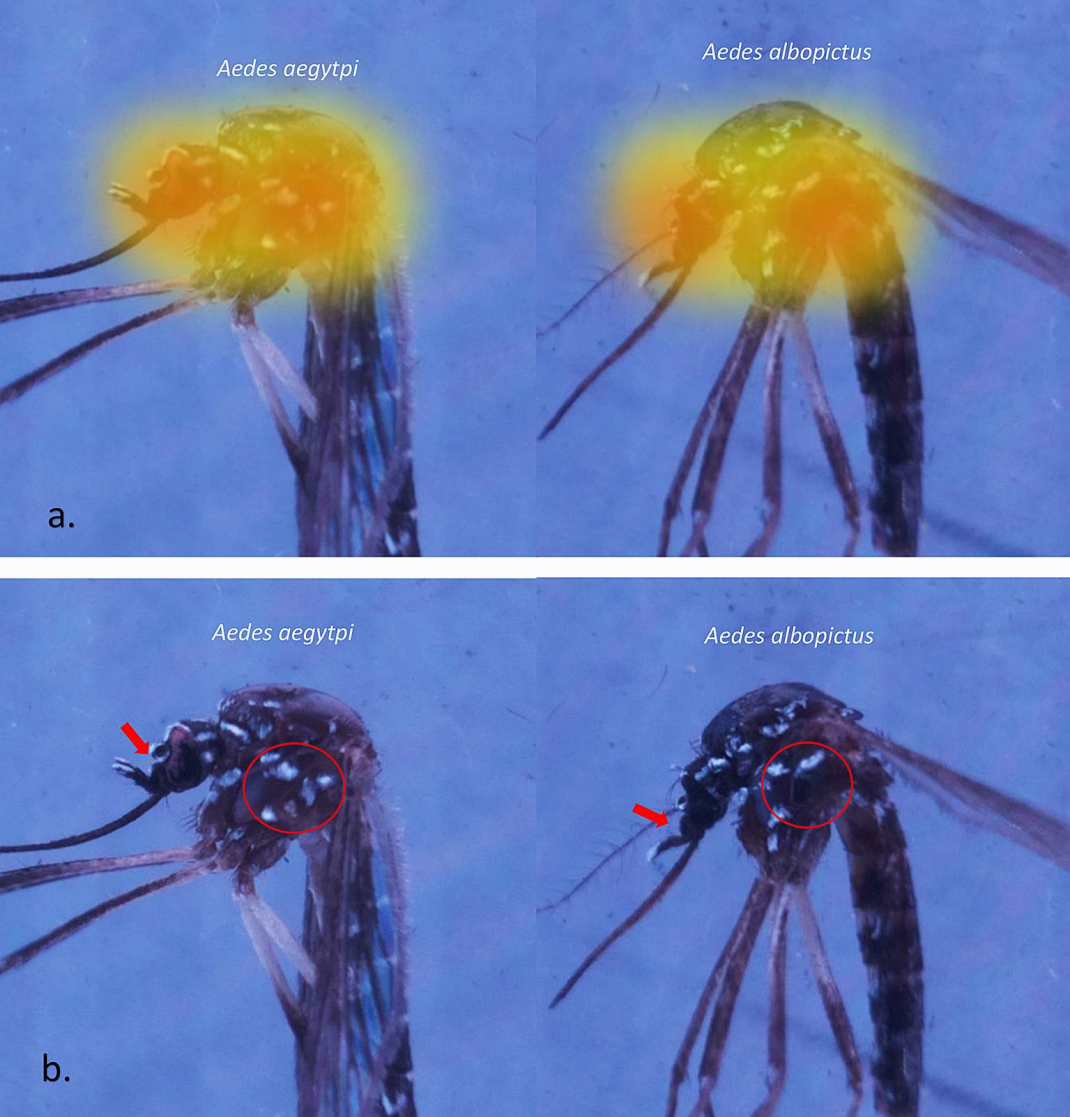
Feature activation on mosquito images. (**a**) Feature activation maps show most of the recognition maps focused on the head and thorax of mosquitoes. (**b**) We suggest that the morphological features used by the DCNN to distinguish between *Aedes aegypti* and *Aedes albopictus* are the mesepimeron, which shows two well-separated white-scale patches (red circles), and the pedicel, which shows white-scale patches (red arrows).

We applied computer vision to the sample mosquitoes to better understand the details of automatic mosquito classification by the DCNNs since most of the previous studies seldom analyzed which parts of mosquitoes were “viewed” and “recognized” by the machine and used to perform classification. Park et al. assessed the feature activation of the convolution layers of VGG-16 and showed that most of the recognition maps focused on the body of mosquitoes. We expanded the idea by designing our labeled image acquisition process to focus mainly on the lateral view of the thorax and head of the mosquitoes since the thorax and head occupied as much as 35% of an image (Figs. 4 and 8), although dorsal and ventral views of the thorax were also contained in the dataset but not as a good morphology of mosquitodue to the disappearance of the white scales. Figure 8 shows a comparison between the key morphology that was used to discriminate the mosquitoes by human experts and the feature activation images of the convolution layers. From the visualization of the convolution layers of our proposed DCNN model, the recognition map focused on the mesepimeron and clypeus-pedicel parts of the *Ae. aegypti* and *Ae. albopictus* images. This result suggested that the distinctive white scales on the pedicel and clypeus (Fig. 8b with red arrow) and the mesepimeron on the *Ae. aegypti*, which are the three types of white scales (Fig. 8b with red circles), are essential to classifying the mosquitoes. When the mosquitoes were 12 to 16 days old, the lyre-shaped markings on *Ae. aegypti* and the white stripe marking on the thorax almost completely disappeared (Fig. 4); however, the scales on the clypeus, pedicel and mesepimeron remained despite the damage to the dorsal or thorax, and these remaining morphologies played crucial roles in differentiating female *Ae. aegypti* from *Ae. albopictus*^30–32^. The improved imageacquisition was attributed to the Aedes Detector, which allowed for a close distance (2.5 cm) and well-illuminated environment, especially regarding the light intensity and distribution. The high accuracy also confirmed the capability of the Pi camera module to acquire the sample images at the necessary resolution, although its resolution was only 8 megapixels.

To obtain the best-performing model, our hyperparameter analysis was in good agreement with previous arguments^25–27^ in which the learning rate (LR) was the key hyperparameter for fine tuning (LR, p < 0.00001), which indicated that the improvement of the model accuracy was more affected by the LR. The LR should not be too small, as mentioned by Goodfellow^33^, and we demonstrated a similar result when the LR of 0.00001 generally produced significantly lower accuracy, with the test accuracy ranging from 93 to 94%. However, higher epochs did not perform significantly higher than lower epochs (Table 1), which may be due to the possibility of higher epochs that led to model overfitting. This was supported by the study of Ladds et al.^34^, which investigated the classification of animal behavior using a super machine learning model and showed that fewer epochs performed better than more epochs in classifying animal behavior. Furthermore, Fabre et al.’s^35^ study tested more epochs, but this decreased the moderately vigorous physical activity and increased the percentage error of the accelerometer measurement. More epoch decreasing the performance of a model could be explained by the possibility of overfitting the model. Nevertheless, our result that shows the lower epochs provided higher accuracy can be explained by the requirement of early stopping of model training at certain epochs, as suggested by Ying^36^, who stated that early stopping at a certain number of epochs can prevent the overfitting of a model.

For an end-to-end process of a model for image classification, Teachable Machine 2.0 by Google Creative Lab is highly convenient in developing a DL model, especially for noncomputer science expertise. The web-based tool assists mostly in the construction of CNN architectures and allowed us to focus on feature engineering and the implementation of the proposed model. To the best of our knowledge, the method is the first reported end-to-end process model for the classification of closely related species of mosquitoes—*Ae. aegypti* and *Ae. albopictus*. To assess the performance of the proposed model, we compared it with human-level performance, in particular, to estimate the optimum or Bayesian error. Human experts classified *Ae. aegypti* and *Ae. albopictus* with an error of ~ 2%, and compared to the error of the proposed model, the model achieved a minimum bias (Bayes error + avoidable error)^37^. A machine learning model with a deep network wants to resemble/surpass human-level performance, and the key aspect of DCNNs is the number of internal layers^38^. We leveraged Teachable Machine 2.0 using transfer learning with DCNNs that adopt the first 29 convolutional layers of MobileNet and reached human expert accuracy. We suggest that the proposed model can surpass human-level performance for non or junior insect taxonomists. Our results extended previous studies that implemented^38,39^ fewer layers of deep convolutional networks (11, 13, 16, and 19 layers) for object recognition but still resembled human-level performance. The implementation accuracy slightly surpassed the human experts' accuracy, although there was no significant difference (independent-samples t-tests, t (58) = 0.297, p = 0.768). As described by Andrew Ng^37^, when an ML model achieves human-level performance, the progress of reducing the desirable error rate will decrease. The automated classification of mosquitoes is mainly attributed to a computer vision system—both the algorithm and the hardware implementation in the present study. Nevertheless, hardware deployment issues such as model drift (when the production data are not representative of the training data), model maintenance and management still posed challenges, especially when the model was applied in a real-time trap or surveillance system, in which regular monitoring and calibration of the model were still required to ensure the validity of the prediction.

## Conclusions

An image classification model for medically important mosquitoes is critical for practical applications in real and actual situations. This study constructed a dataset of 12- to 16-day-old *Ae. aegypti* and *Ae. albopictus* with disappearances of the head and thorax morphologies. The model was successfully developed using Aedes Detector hardware, and the results indicated the importance of the environment to obtain images for computer vision and therefore constructed a dataset and model that was used to infer unseen samples. We demonstrated the capacity of the proposed model with a DCNN to classify aged mosquitoes with more than 98% testing accuracy, with both the learning rate and epochs significantly influencing the model performance. Most strikingly, when the model was deployed externally on three different generations of mosquitoes, the accuracy resembled that of human experts. Our study provides a framework for future studies that can utilize the proposed method to assess the quality of data and training conditions, statistically study hyperparameters, and implement hardware on an external sample.

## Data Availability

The images, dataset and source codes for this work are publicly available through the co-author (PI)’s kaggle: https://www.kaggle.com/pradeepisawasan/aedes-mosquitos.

## References

[1] WHO. *Dengue and Severe Dengue* (April 2020).

[2] Alongkot P, Jeffrey GS, Laura CH (2005). Insecticide Susceptibility of *Aedes aegypti* and *Aedes albopictus* across Thailand. J. Med. Entomol..

[3] Kweka EJ, Mahande AM (2009). Comparative evaluation of four mosquitoes sampling methods in rice irrigation schemes of lower Moshi, northern Tanzania. Malar. J..

[4] Servsadse M (2012). Medical Entomology for Students.

[5] Silva, D. F., Souza, V. M. A. D., Batista, G. E. A. P. A., Keogh, E., & Ellis, D. P. W. Applying machine learning and audio analysis techniques to insect recognition in intelligent traps. In 2013 *12th International Conference on Machine Learning and Applications, Miami, FL*. 99–104 (2013).

[6] De Los Reyes, A. M. M., Reyes, A. C. A., Torres, J. L., Padilla, D. A. and Villaverde, J. Detection of *Aedes aegypti* mosquito by digital image processing techniques and support vector machine. In 2016 *IEEE Region 10 Conference (TENCON), Singapore*. 2342–2345 (2016).

[7] Mulchandani P, Siddiqui MU, Kanani P (2000). Real-time mosquito species identification using deep learning techniques. Int. J. Eng. Adv. Technol..

[8] Xia D, Chen P, Wang B, Zhang J, Xie C (2018). Insect detection and classification based on an improved convolutional neural network. Sensors..

[9] Mohd Fuad MA (2019). Detection of *Aedes aegypti* larvae using single shot multibox detector with transfer learning. Bull. Electric. Eng. Inf..

[10] Okayasu K, Yoshida K, Fuchida M, Nakamura A (2019). Vision-based classification of mosquito species: Comparison of conventional and deep learning methods. Appl. Sci..

[11] Maciel-de-Freitas R (2014). Discrepancies between *Aedes aegypti* identification in the field and in the laboratory after collection with a sticky trap. Mem. Inst. Oswaldo Cruz..

[12] Shameem Fathima A, Manimegalai D, Hundewale N (2011). A review of data mining classification techniques applied for diagnosis and prognosis of the arbovirus-dengue. IJCSI Int. J. Comput. Sci..

[13] Mona, M. A machine learning framework to classify mosquito species from smart-phone images. *Graduate Theses and Dissertations*. https://scholarcommons.usf.edu/etd/7340 (2018).

[14] Goodwin A (2020). Development of a low-cost imaging system for remote mosquito surveillance. Biomed. Opt. Express..

[15] Motta D (2019). Application of convolutional neural networks for classification of adult mosquitoes in the field. PLoS ONE.

[16] Park J (2020). Classification and morphological analysis of vector mosquitoes using deep convolutional neural networks. Sci. Rep..

[17] Sanjiv, K.B. *et al.* Advances in computer communication and computational sciences. *Proceedings of IC4S*. **1**, (Springer, 2017)

[18] García-Martín E (2019). Estimation of energy consumption in machine learning. J. Parallel Distrib. Commun..

[19] Maryam MN, Villanustre F, Khoshgoftaar TM, Seliya N, Wald R, Muharemagic E (2015). Deep learning applications and challenges in big data analytics. J. Big. Data..

[20] Blier, L., Wolinski, P., & Ollivier, Y. *Learning with Random Learning Rates*. (ed. Brefeld U., Fromont E., Hotho A., Knobbe A., Maathuis M., Robardet C. (Springer, 020)

[21] Reitermanov, Z. Data Splitting. *WDS'10 Proceedings of Contributed Papers*, Part I, 31–36, (2010).

[22] Qi, H., Liu, W. & Liu, L. An efficient deep learning hashing neural network for mobile visual search. In 2017 *IEEE Global Conference on Signal and Information Processing (GlobalSIP)* (2017)

[23] Howard *et al.**MobileNets: Efficient Convolutional Neural Networks for Mobile Vision Applications*. https://arxiv.org/pdf/1704.04861.pdf (2017).

[24] Tang G (2019). Improved convolutional neural networks for acoustic event classification. Multimed. Tools Appl..

[25] Wang, L., Dernoncourt, F. & Bui, T. Bayesian optimization for selecting efficient machine learning models. In *CIKM 2019 MoST-Rec Workshop* (2019)

[26] Fabre N, Lhuisset L, Bernal C, Bois J (2020). Effect of epoch length on intensity classification and on accuracy of measurement under controlled conditions on treadmill: Towards a better understanding of accelerometer measurement. PLoS ONE.

[27] Gupta, S., Zhang, W., Wang, F. Model accuracy and runtime tradeoff in distributed deep learning: A systematic study. In 2017 *Proceedings of the Twenty-Sixth International Joint Conference on Artificial Intelligence (IJCAI-17)* (2017)

[28] Google Developers. Descending into ML: Training and loss. *Machine Learning Crash Course*. https://developers.google.com/machine-learning/crash-course/descending-into-ml/training-and-loss (2020)

[29] Khalifa NEM, Loey M, Taha MHN (2020). Insect pests recognition based on deep transfer learning models. J. Theor. Appl. Inf. Technol..

[30] Bar A, Andrew J (2013). Morphology and morphometry of *Aedes aegypti* adult mosquito. Annu. Res. Rev. Biol..

[31] Savage HM, Smith GC (1995). *Aedes albopictus* y *Aedes aegypti* en las Américas: Implicaciones para la transmisión de arbovirus e identificación de hembras adultas dañadas” [*Aedes albopictus* and *Aedes aegypti* in the Americas: Implications for the transmission of arboviruses and identification of damaged adult females]. Bol. Oficina Sanit. Panam..

[32] Buxton M, Lebani K, Nyamukondiwa C, Wasserman RJ (2019). First record of *Aedes* (Stegomyia) *aegypti* (Linnaeus, 1762) (Diptera: Culicidae) in Botswana. Bioinvas. Rec..

[33] Goodfellow, I., Bengio, Y., Courville, A. *Deep Learning*. (MIT Press, 2016). http://www.deeplearningbook.org.

[34] Ladds MA, Thompson AP, Kadar J (2017). Super machine learning: improving accuracy and reducing variance of behaviour classification from accelerometry. Anim. Biotelemet..

[35] Fabre N, Lhuisset L, Bernal C, Bois J (2020). Effect of epoch length on intensity classification and on accuracy of measurement under controlled conditions on treadmill: Towards a better understanding of accelerometer measurement. PLoS ONE.

[36] Ying X (2019). An overview of overfitting and its solutions. J. Phys. Conf. Ser..

[37] Andrew, N. Machinelearning yearning: Technical strategy for AI engineers, in the era of deep learning. Deeplearning.ai (2018)

[38] Kheradpisheh S, Ghodrati M, Ganjtabesh M (2016). Deep networks can resemble human feed-forward vision in invariant object recognition. Sci. Rep..

[39] Simonyan, K. & Zisserman, A. Very deep convolutional networks for large-scale image recognition. https://arxiv.org/pdf/1409.1556.pdf (2014).

